# Selected cytokine and chemokine concentrations in equine autologous conditioned serum are similar under defined and practically relevant storage conditions

**DOI:** 10.3389/fvets.2025.1588240

**Published:** 2025-05-27

**Authors:** Susanne Pauline Roth, Giulia Liso, Walter Brehm, Bettina Wagner, Christiane Liliane Schnabel, Antonia Troillet

**Affiliations:** ^1^Veterinary Teaching Hospital, Department for Horses, Faculty of Veterinary Medicine, Leipzig University, Leipzig, Germany; ^2^Saxonian Incubator for Clinical Translation (SIKT), Leipzig University, Leipzig, Germany; ^3^Department of Population Medicine and Diagnostic Sciences, College of Veterinary Medicine, Cornell University, Ithaca, NY, United States; ^4^Institute of Immunology, Faculty of Veterinary Medicine, Leipzig University, Leipzig, Germany

**Keywords:** orthobiologics, autologous blood products, blood cell secretome, monoclonal antibody, multiplex assay, orthopedics, horse

## Abstract

**Background:**

The anti-inflammatory and regenerative effects of autologous conditioned serum (ACS) in joint-associated disorders are presumably mediated by the biomolecules it contains, such as cytokines and chemokines. ACS is commonly used in equine practice after storage. Data regarding the influence of storage conditions of ACS on cytokine and chemokine concentrations are not available.

**Objectives:**

To evaluate the effect of commonly used storage conditions on selected cytokine and chemokine concentrations in equine ACS.

**Methods:**

From 10 horse patients with clinically indicated ACS preparation six ACS aliquots each were stored under different storage conditions. The samples were evaluated after storage at room temperature (rt) or at refrigerator temperature (6–8°C) for 6 h. Another set of samples was stored frozen at −20°C for 7 months. After this storage under clinically relevant conditions all samples were frozen at −80°C, and compared to an aliquot immediately frozen at −80°C. Mediator concentrations were determined by fluorescent bead-based multiplex assays using monoclonal antibodies that are specific for the respective analyzed molecule: interferon (IFN)-γ, interleukin (IL)-17A, IL-4, IL-10, and IFN-α (equine cytokine 5-plex assay) as well as CC-chemokine ligand 2 (CCL2), CCL3, CCL5, CCL11, tumor necrosis factor (TNF)-α, and IL-1β (equine chemokine 6-plex assay) were analyzed.

**Results:**

Concentrations of IFN-γ and IL-10, as well as the chemokines analyzed, showed marked interindividual differences. The cytokines IL-17A, IL-4, and IFN-α were not detected in any of the samples analyzed. Concentrations of selected cytokines, and chemokines were similar between the defined storage conditions.

**Conclusions:**

Concentrations of selected cytokines, chemokines, and IL-1ra in equine ACS were not influenced by the defined storage conditions studied (rt, refrigerated, frozen).

## 1 Introduction

Autologous conditioned serum (ACS) is a blood product composed of serum and the blood cell secretome after extended coagulation. This orthobiological therapeutic agent, widely used to treat equine inflammatory orthopedic conditions (1–3), is prepared individually for each patient according to the medicinal product manufacturer's guidelines, which include a defined incubation period at 37°C, and a centrifugation cycle. In addition to medical products approved for veterinary [(ABPS—Autologous Blood Processing System 60 ml; Arthrex^®^ VetSystems, Arthrex GmbH); (Orthokine^®^ vet irap 10/60 ml; ORTHOGEN Veterinary GmbH)] (4–18) and human medicine [(EmCyte Corporation); (Onoccomed; Plasmaconcept AG)] (19), the current literature also describes non-commercial methods (incubated serum from glass or non-glass tubes) for equine ACS production (5, 8, 15, 20, 21). Approved medical products generally contain medicinal-grade glass beads, which are not used in non-commercial manufacturing methods. The incubation of whole blood takes place at 37°C, the incubation time varies according to the manufacturer depending on the blood volume (6–8 or 24 h) or from 12 to 36 h for non-commercial manufacturing methods. Information on the subsequent centrifugation varies despite detailed specifications of the medical product manufacturers [the often-given unit rpm (revolutions per minute) cannot be converted into the standard rcf (relative centrifugal force) without the rotor diameter of the centrifuge used].

The clinically observed effects of ACS cannot be attributed to a single component, such as interleukin-1 receptor antagonist (IL-1ra), whose increased production by autologous blood as an IL-1 inhibitor was the original idea behind the development of ACS as a therapeutic agent (22, 23). Rather, the mechanism of action of ACS in producing significant anti-inflammatory effects and potential tissue regeneration appears to be multifaceted. Current literature focuses primarily on data obtained from enzyme-linked immunosorbent assays (ELISA) (4, 6–8, 10, 11, 19, 21) and multiplex assays (5, 9, 20) that quantify protein levels involved in regulating pro- and anti-inflammatory responses (cytokines), cellular proliferation and differentiation (growth factors), and cell migration and recruitment (chemokines). Inconsistent biomolecule detection methods and ACS manufacturing protocols limit the comparability of results and final conclusions.

In addition, different study populations may influence ACS composition. Patient-related endogenous factors, although still unclear, have been widely demonstrated to result in large interindividual differences in the concentration of some cytokines in baseline and ACS samples (5, 6, 8, 10, 11, 19, 20). Furthermore, the endogenous control of cytokine and growth factor release by neuroendocrine mechanisms leads to fluctuations in circulating concentrations with a diurnal rhythm (24–26). Also, vaccinations or viral infections affect serum concentrations of cytokines such as interferon (IFN)-γ (27, 28). In addition, possible patient-related exogenous factors influencing equine ACS biomolecule levels include surgical stress and exhaustive exercise (19, 20).

Exogenous handling influences on ACS, such as storage time and temperature, have been little studied. However, human cytokines, like other blood proteins, can be degraded or be affected by storage and transport conditions, with endogenous and recombinant cytokines being susceptible to varying degrees (29–31). To the best of the author's knowledge, there is only one study on the storage conditions of equine ACS (5). Hale et al. (5) reported that in contrast to IL-10, IL-1β, and tumor necrosis factor (TNF)-α, which were stable during five freeze-thaw cycles, IL-1ra concentrations decreased with increasing number of freeze-thaw cycles, significantly after the fifth freeze-thaw cycle.

The stability of mediators relevant for the therapeutic effect of ACS during storage is of critical importance because in routine clinical practice, a delay between ACS preparation and administration is often unavoidable. This is due to various scenarios, such as the travel time in ambulatory settings, hospital workflows that prevent immediate ACS application, or cases where multiple injections are planned for repeated treatments. Therefore, the objective of this study was to determine the potential effect of storage conditions mimicking clinical practice situations on cytokine, chemokine, and IL-1ra concentrations in ACS. It was hypothesized that the concentrations of selected cytokines, chemokines, and of IL-1ra in equine ACS would not be affected by prolonged storage at room or refrigerator temperature either immediately after ACS preparation or after an additional 7 months of frozen storage (28 weeks).

## 2 Materials and methods

### 2.1 Horses

The animal study was reviewed and approved by the internal faculty Ethics Committee (approval number EK 21/2023). Written consent was obtained from the owners for the participation of their animals in this study. Ten adult horses presented to the clinic for treatment with ACS were included in the study. The mean [± standard deviation (SD)] age was 10.7 (± SD 5.1) years (range 3–17 years), all horses were female. Breeds included Warmbloods (*n* = 6), Draft horses (*n* = 2), and Icelandic horses (*n* = 2). The mean (± SD) bodyweight of the mares was 511.2 (± SD 97.67) kg (range 407–694 kg). ACS treatment was indicated due to orthopedic diseases affecting either the fetlock joint, the talocrural joint, the digital flexor tendon sheath, the inferior check ligament, the proximal suspensory ligament, or the plantar annular ligament (Supplementary Table S1). Included horses had vital signs within physiological ranges at the time of venipuncture for ACS preparation. There was no control of blood parameters at the time of venipuncture for ACS preparation. The time for venipuncture indicated for ACS preparation was similar for all included horses (00:00 and 02:30 a.m.; except for one horse at 11:10 a.m.). Included horses did not perform any prolonged exercise. Surgery was performed on six out of 10 horses between 48 h and 7 days prior to venipuncture for ACS preparation. No sedation was used within 24 h of venipuncture for ACS preparation in six out of 10 horses; sedation was used 12 and 24 h before venipuncture for ACS preparation in three out of 10 horses and one out of 10 horses, respectively. Blocking was not performed in nine out of 10 horses, one horse was blocked 12 h prior to venipuncture for ACS preparation.

### 2.2 Sample collection

During the venipuncture indicated for ACS preparation, additional 20 ml of whole blood were collected from each of the 10 horses. Blood collection was performed under sterile conditions from the jugular vein. After checking both jugular veins for clinical abnormalities, one jugular vein was chosen individually according to clinical and practical criteria. At the puncture site, hairs were clipped, and the skin underwent antiseptic skin preparation [scrub with antibacterial liquid soap containing polyvinylpyrrolidone (PVP) iodine and 96% ethanol (Jodosept^®^ PVP; Vetoquinol GmbH), 70% ethanol (2-Propanol 70%; B. Braun SE), and 70% isopropyl alcohol and povidone iodine solution (Braunoderm^®^ B. Braun SE)]. Wearing sterile gloves, the blood was collected using a 21 G safety winged intravenous needle (Venofix^®^ Safety; B. Braun SE) and a registered medicinal product for ACS preparation (Orthokine^®^ vet irap 10 ml; ORTHOGEN Veterinary GmbH). Detached syringes (twice 10 ml each horse) filled with whole blood were gently inverted several times and processed according to the manufacturer's instructions (incubation at 37°C for 7 h, centrifugation at 3,000 × *g* for 10 min, aspiration of ACS) (32). Aspirated ACS was portioned in six aliquots (1.2–2.2 ml each) in sterile 3 ml capped syringes (3 ml HENKE-JECT^®^ Henke-Sass, Wolf GmbH; Combi-Stopper red; B.Braun SE).

### 2.3 Storage testing

**Figure 1:**
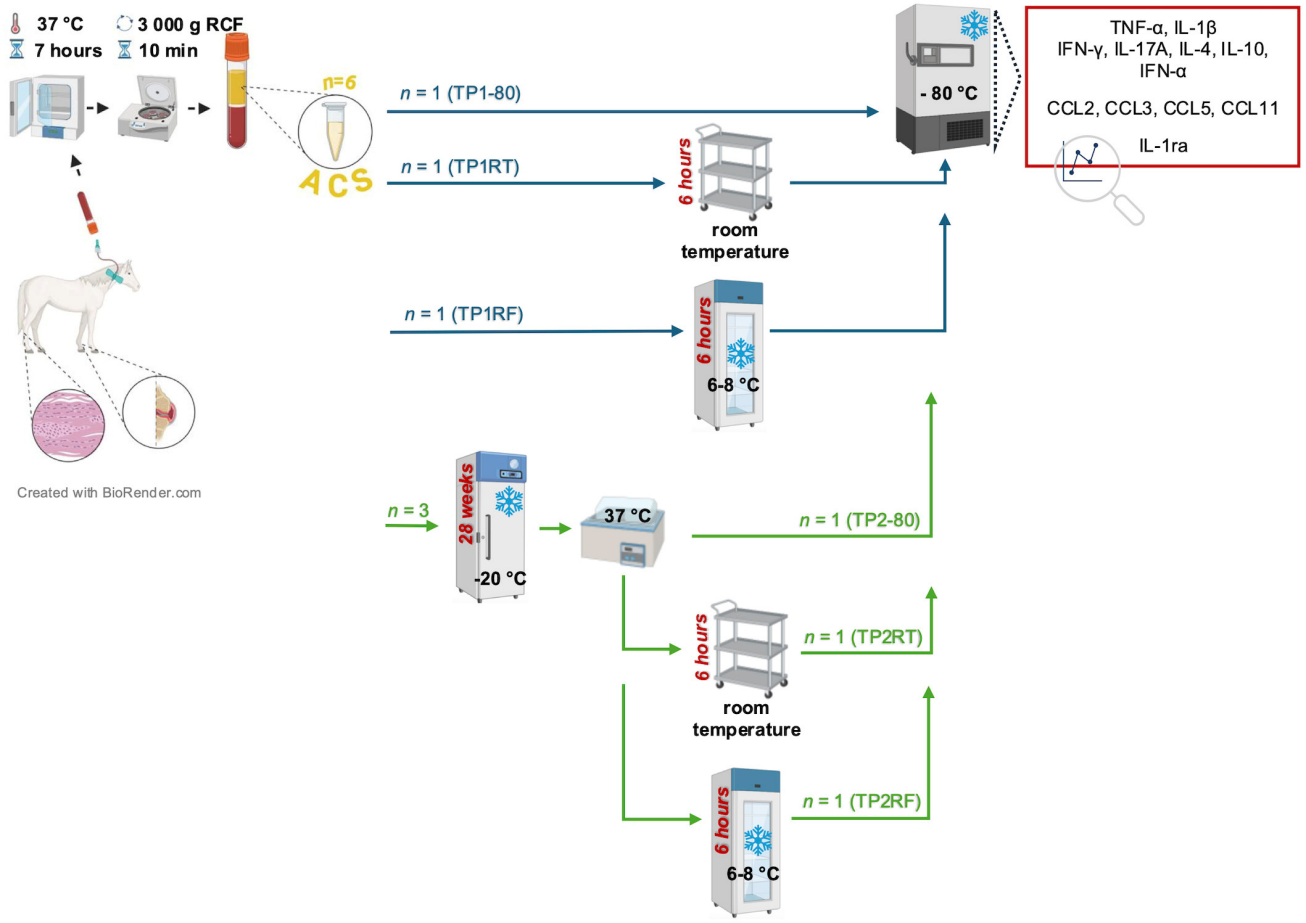
Schematic overview of the study design. Autologous conditioned serum (ACS) samples (*n* = 6) per horse (*n* = 10) were prepared by incubation of venous whole blood at 37°C for 7 h and subsequent centrifugation (3,000*g* RCF for 10 min). Prepared ACS samples were labeled with respect to their storage treatment as the following treatment groups: treatment groups of time point 1 (TP1-80, TP1RT, TP1RF) were stored either immediately at −80°C (TP1-80, basal ACS control) or were stored for 6 h either at room temperature (TP1RT) or at refrigerator temperature at 6–8°C (TP1RF) before being stored at −80°C; treatment groups of time point 2 (TP2-80, TP2RT, TP2RF) underwent a frozen storage at −20°C for 7 months (28 weeks) before they were thawed at a water bath (37°C), and were then either immediately stored at −80°C (TP2-80) or were stored for 6 h either at room temperature (TP2RT) or at a refrigerator temperature at 6–8°C (TP2RF). At the end of their respective storage time, TP2RT and TP2RF also were stored at −80°C before further analysis. Concentrations of selected cytokines (TNF-α, IL-1β IFN-γ, IL-17A, IL-4, IL-10, and IFN-α) and chemokines (CCL2, CCL3, CCL5, and CCL11) were quantified by fluorescent bead-based multiplex assays in all six ACS samples per horse.

A schematic overview for the performed storage testing is given in Figure 1. In 10 adult horses that were presented for a therapeutic ACS application, additional six ACS samples were prepared and labeled with respect to their storage treatment as the following treatment groups: TP1-80 (time point 1: immediate storage at −80°C); TP1RT (time point 1: 6 h storage at room temperature); TP1RF (time point 1: 6 h storage at refrigerator temperature); TP2-80 (time point 2: immediate storage at −80°C); TP2RT (time point 2: 6 h storage at room temperature); and TP2RF (time point 2: 6 h storage at refrigerator temperature). Time point 1 (TP1) was set immediately after ACS preparation, and time point 2 (TP2) was set after a 7 month (28 weeks) of storage at −20°C and thawing in a water bath (37°C). After the storage testing was completed on all treatment groups from the 10 horses, all ACS samples underwent an initial slow thaw process for re-aliquoting and were again stored at −80°C. To enable cytokine, chemokine, and IL-1ra quantification, frozen ACS samples were shipped on dry ice and arrived at their destination frozen.

### 2.4 Multiplex assay

All samples were analyzed by the equine cytokine 5-plex assay for interferon (IFN)-γ, IL-17A, IL-4, IL-10, and IFN-α, and by the equine chemokine 6-plex assay for CC-chemokine ligand (CCL)2, CCL3, CCL5, CCL11, tumor necrosis factor (TNF)-α, and IL-1β (Animal Health Diagnostic Center at Cornell University College of Veterinary Medicine, USA). These fluorescent bead-based multiplex assays quantify the respective target molecules simultaneously by using pairs of equine specific monoclonal antibodies (33–35).

### 2.5 IL-1ra ELISA

All samples were analyzed in duplicates by the Equine IL-1ra/IL-1F3 DuoSet ELISA (R&D Systems, Inc.) according to the manufacturer's recommendations using plates and buffers from a DuoSet Ancillary Reagent Kit (R&D Systems, Inc.). The plates were coated with the polyclonal capture antibody provided in phosphate buffered saline (PBS), washed with PBS-T [PBS, 0.05% v/v Tween20 (Carl Roth GmbH + Co. KG)], and blocked with reagent diluent (PBS containing 1% v/v bovine serum albumin), and washed again. The IL-1ra standard was prepared as a serial dilution (0.3125–20 ng/ml) diluted in normal goat serum (R&D Systems, Inc.). Diluted normal goat serum was also used as blanks to mimic a serum-comparable assay matrix. Samples were diluted 1:10 in reagent diluent. After incubation with standards/blanks/samples and another washing step, the polyclonal detection antibody, followed by washing, Streptavidin-HRP, washing, TMB substrate, and Stop solution were added according to the manufacturer's instructions. Optical densities at 450 nm, corrected by those at 520 and 570 nm were recorded on a SpectraMax^®^ 340 instrument with Softmax Pro software (Molecular Devices, Thermo Fisher Scientific Inc.). Averaged blank-reduced optical densities were used to interpolate a standard curve and concentrations via a sigmoidal 4-PL approach using Prism version 10.3.0 (GraphPad Software by Dotmatics).

### 2.6 Data analysis

Statistical data analysis was conducted with Prism version 10.3.0 (GraphPad Software by Dotmatics). Descriptive statistical analyses of measured cytokine and chemokine concentrations included the calculation of mean, standard deviation (SD), median, and interquartile range. Normal distribution tests (Shapiro–Wilk test) were performed to determine the degree of skewness. To examine differences in cytokine and chemokine concentrations of all ACS samples, the Friedman test was used as a rank-based non-parametric test to analyze repeated measures data. Subsequent multiple comparisons within each treatment group (TP1-80, TP1RT, TP1RF, TP2-80, TP2RT, and TP2RF) were performed using Dunn's multiple comparison test (15 comparisons per treatment group). A *p*-value of < 0.05 was used as the threshold for statistical significance.

## 3 Results

### 3.1 Storage conditions do not affect selected cytokine concentrations in equine ACS

**Figure 2:**
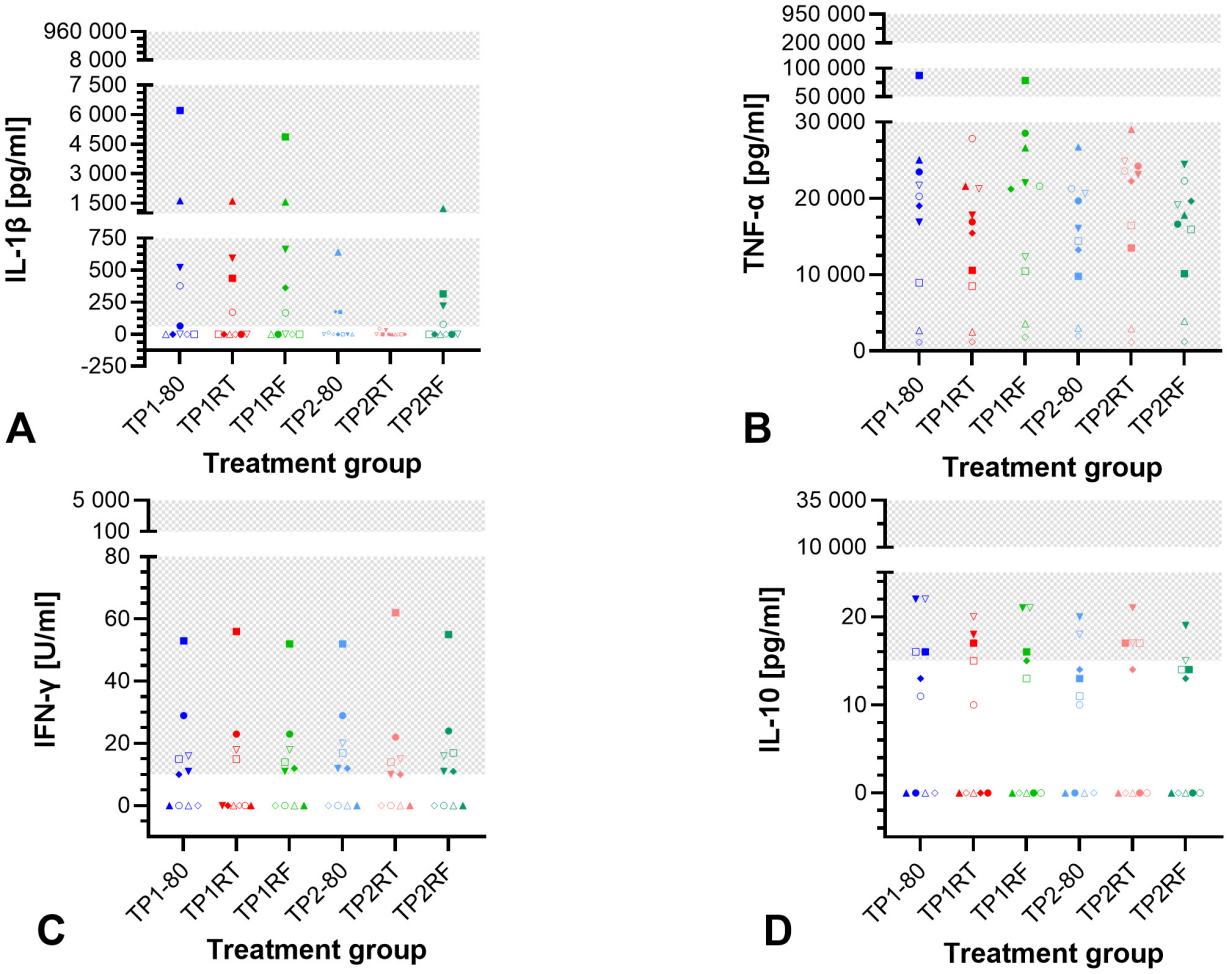
Dot plots showing cytokine concentrations in the six autologous conditioned serum (ACS) treatment groups. The concentrations of IL-1β (pg/ml) **(A)**, TNF-α (pg/ml) **(B)**, IFN-γ (U/ml) **(C)**, and IL-10 (pg/ml) **(D)** are given for ACS samples from 10 horses after six different storage conditions each. IL-17A, IL-4, and IFN-α were not detected in any ACS sample. Treatment group TP1-80 is the basal ACS control (time point 1: immediate storage at −80°C), TP1RT (time point 1: 6 h storage at room temperature) was stored at room temperature for 6 h, TP1RF (time point 1: 6 h storage at refrigerator temperature) was stored at refrigerator temperature at 6–8°C for 6 h; treatment groups TP2-80, TP2RT, and TP2RF were stored at −20°C for 7 month (28 weeks; time point 2) before they were thawed at a water bath (37°C), and then either immediately were stored at −80°C (TP2-80) or were stored for 6 h either at room temperature (TP2RT) or at refrigerator temperature at 6–8°C (TP2RF). The horses are indicated by symbols: horse 1 ◦, horse 2 □, horse 3 Δ, horse 4 ▽, horse 5 ♢, horse 6 ●, horse 7 ■, horse 8 ▴, horse 9 ▾, horse 10 ♦. The gray checkered areas indicate the detection range of the respective multiplex assay (IL-1β: 61–960,000 pg/ml; TNF-α: 61–950,000 pg/ml; IFN-γ: 10–5,000 U/ml; IL-10: 15–35,000 pg/ml). Cytokines are reported in pg/ml, except for IFN-γ reported in U/ml. All data were not normally distributed. There are no statistically significant differences between the different storage conditions for IL-1β **(A)**, TNF-α **(B)**, IFN-γ **(C)**, and IL-10 **(D)** concentrations (*p* > 0.05).

For IL-1β, Friedman test showed a significant difference (*p* = 0.0048), but after Dunn's multiple comparisons test, the required level of significance (*p* < 0.05) was no longer met (Figure 2A). IL-1β was undetectable in any samples from four horses, the remaining six horses had at least one sample without IL-1β or IL-1β below the detection limit. The concentrations of TNF-α, IFN-γ, and IL-10 were not significantly different between different storage conditions (*p* = 0.058, *p* = 0.166, and *p* = 0.132, respectively in Friedman tests, Figures 2B–D). TNF-α concentrations were within the detection limit in all samples. IFN-γ concentrations were below the detection limit in each sample from four horses and in treatment group TP1RT from two horses. Also, only two horses had IL-10 concentrations above the lower detection limit in each sample. In samples from four horses, IL-10 was not detected in any sample. Median values and interquartile ranges for IL-1β, TNF-α, IFN-γ, and IL-10 concentrations are listed in Supplementary Table S2. IL-17A, IL-4, and IFN-α were not detectable in any sample.

### 3.2 Storage conditions do not affect chemokine concentrations in equine ACS

**Figure 3:**
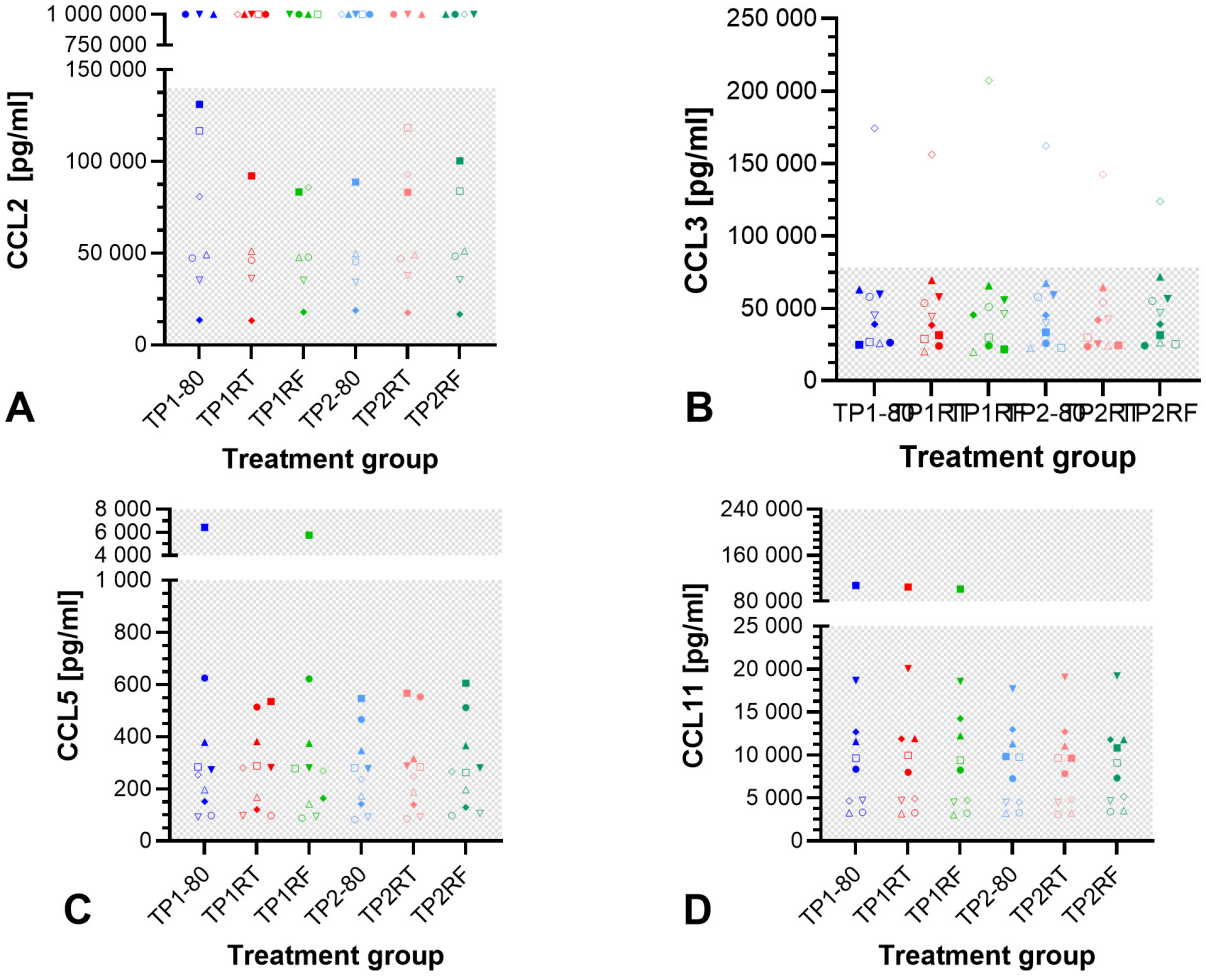
Dot plots showing chemokine concentrations in the six autologous conditioned serum (ACS) samples. The concentrations of CCL2 (pg/ml) **(A)**, CCL3 (pg/ml) **(B)**, CCL5 (pg/ml) **(C)**, and CCL11 (pg/ml) **(D)** are given for ACS samples from 10 horses after six different storage conditions each. Treatment group TP1-80 is the basal ACS control (time point 1: immediate storage at −80°C), TP1RT (time point 1: 6 h storage at room temperature) was stored at room temperature for 6 h, TP1RF (time point 1: 6 h storage at refrigerator temperature) was stored at refrigerator temperature at 6–8°C for 6 h; treatment groups TP2-80, TP2RT, and TP2RF were stored at −20°C for 7 month (28 weeks) (time point 2) before they were thawed at a water bath (37°C), and then either immediately were stored at −80°C (TP2-80) or were stored for 6 h either at room temperature (TP2RT) or at refrigerator temperature at 6–8°C (TP2RF). The horses are indicated by symbols: horse 1 ◦, horse 2 □, horse 3 Δ, horse 4 ▽, horse 5 ♢, horse 6 ●, horse 7 ■, horse 8 ▴, horse 9 ▾, horse 10 ♦. The gray checkered areas indicate the detection range of the respective multiplex assay (CCL2: 9–140,000 pg/ml; CCL3: 5–78,242 pg/ml; CCL5: 5–77,000 pg/ml; CCL11: 18–280,000 pg/ml). All data were not normally distributed. There are no statistically significant differences between the different storage conditions for CCL2 **(A)**, CCL3 **(B)**, CCL5 **(C)**, and CCL11 **(D)** concentrations (*p* > 0.05). The symbols at a CCL2 concentration of 1,000,000.00 pg/ml **(A)** indicate a measured value that was above the upper limit of detection (>140,000 pg/ml).

The concentrations of CCL2, CCL3, CCL5, and CCL11 were not significantly different between the different storage conditions (*p* = 0.736, *p* = 0.372, *p* = 0.103, and *p* = 0.135, respectively in Friedman tests, Figure 3). CCL2 concentrations exceeded the detection range (concentration set to 1,000,000 pg/ml) in all samples from three horses, and in three individual samples from two horses. The remaining CCL2 concentrations were within the detection range. For CCL3, one horse had concentrations above the upper limit of detection measured in all the six samples. For CCL5 and CCL11, all measured concentrations were within the detection range, with one horse's samples in the upper CCL2 end of the range for the treatment groups TP1-80 and TP1RF (CCL5) and for the treatment groups TP1-80, TP1RT, and TP1RF (CCL11). Overall, chemokine concentrations showed marked interindividual differences between the 10 horses. Median values and interquartile ranges for CCL2, CCL3, CCL5, and CCL11 concentrations are listed in Supplementary Table S2.

### 3.3 The defined storage conditions do not affect IL-1ra concentrations in equine ACS

**Figure 4:**
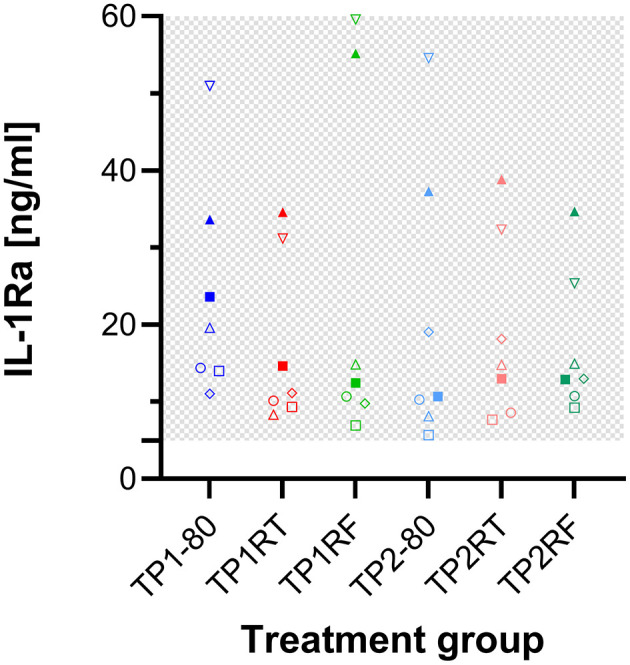
Dot plots showing IL-1ra concentrations in the six autologous conditioned serum (ACS) samples. The concentrations of IL-1ra (ng/ml) are given for ACS samples from seven horses after six different storage conditions each. IL-1ra concentrations for ACS of the remaining three horses exceeded the detection range and are therefore not shown here. Treatment group TP1-80 is the basal ACS control (time point 1: immediate storage at −80°C), TP1RT (time point 1: 6 h storage at room temperature) was stored at room temperature for 6 h, TP1RF (time point 1: 6 h storage at refrigerator temperature) was stored at refrigerator temperature at 6–8°C for 6 h; treatment groups TP2-80, TP2RT, and TP2RF were stored at −20°C for 7 month (28 weeks; time point 2) before they were thawed at a water bath (37°C), and then either immediately were stored at −80°C (TP2-80) or were stored for 6 h either at room temperature (TP2RT) or at refrigerator temperature at 6–8°C (TP2RF). The horses are indicated by symbols: horse 1 ◦, horse 2 □, horse 3 Δ, horse 4 ▽, horse 5 ♢, horse 6 ●, horse 7 ■, horse 8 ▴, horse 9 ▾, horse 10 ♦. The gray checkered areas indicate the detection range of the ELISA. All data were not normally distributed. The IL-1ra concentrations of the seven horses were similar between the different storage conditions. The precise IL-1ra concentrations in ACS warrant some caution since their quantification may not be accurate due to matrix effects on the ELISA.

The concentrations of IL-1ra in all ACS samples from three horses exceeded the detection range. Those from the seven horses were in range, displayed marked interindividual differences, but were similar between the different storage conditions (Figure 4). Of note, higher dilution (20 instead of 10) of the samples did not yield plausible values and the quantification of IL-1ra concentrations in ACS may not be accurate due to matrix effects on the ELISA.

## 4 Discussion

The hypothesis of the present study that selected cytokine, chemokine, and IL-1ra concentrations in equine ACS remain stable after storage of ACS for 6 h at room or refrigerator temperature either immediately after preparation or after 7 months (28 weeks) at −20°C was proven. Our present study revealed no significant difference in selected cytokine, chemokine, and IL-1ra concentrations in equine ACS between samples under different storage conditions [ACS storage for 6 h at room or refrigerator temperature either immediately after preparation or after 7 months (28 weeks) at −20°C]. Of the 12 biomolecule concentrations measured IL-17A, IL-4, and IFN-α were not detectable in any of the ACS samples. Concentrations of the remaining nine biomolecules (cytokines IL-1β, TNF-α, IFN-γ, IL-10; chemokines CCL2, CCL3, CCL5, CCL11; IL-1ra) showed clear interindividual differences.

The storage testing used was chosen to mimic conditions under which ACS is used in equine clinical practice. ACS applications are typically used for therapeutic purposes fresh after production (treatment group TP1-80) or application is delayed for other reasons and therefore stored for several hours at room temperature (treatment group TP1RT) or in the refrigerator (treatment group TP1RF). The selected storage time of 6 h at room or refrigerator temperature was chosen, to allow sufficient time for preparation of ACS in the morning (i.e., at the start of the working day) and subsequent application at lunchtime, as this is a realistic condition for ambulatory work. According to the manufacturer's recommendation it is also possible to use ACS therapeutically after a maximum storage period of 12 months at −18°C, e.g., for repeated applications (32). Accordingly, the ACS can be applied directly after being thawed (treatment group TP2-80) or after several hours of storage at room temperature (treatment group TP2RT) or in the refrigerator (treatment group TP2RF) due to reasons of clinical practicability. The selected storage period of 7 months at −20°C covers the recommended regimen of one to four injections at 8–14 days intervals (depending on the respective indication, as per the manufacturer's instructions) (32), and furthermore repeated administrations for relapses up to 7 months after initial disease onset. The extended storage of 7 months at −20°C was chosen, since from the author's clinical point of view, a relapse within a time frame longer than 6–7 months is less likely. Because freezing at −80°C necessitates specific laboratory freezing systems, this storage method is not commonly available for clinical practitioners. ACS was prepared using a commercially available medicinal product (Orthokine^®^ vet irap 10 ml; ORTHOGEN Veterinary GmbH), that is approved for use in veterinary medicine. There was no comparison with any other medicinal product for ACS preparation. Concentrations of selected cytokines and chemokines were analyzed by fluorescent bead-based multiplex assays as, to the best of the author's knowledge, these are the only validated assays using pairs of equine specific monoclonal antibodies. IL-1ra concentrations were measured using a polyclonal antibody-based ELISA, as there is no validated equine-specific assay for IL-1ra quantification in blood products.

The exact composition of equine ACS is still unknown. The present study measured in total 12 biomolecules from which IL-17A, IL-4, and IFN-α were not detectable in any ACS sample. To the best of the author's knowledge, all three cytokines have not previously been measured in equine ACS. In human ACS, IL-4 has been measured by ELISA and was significantly enriched after a 24-h incubation compared to serum (analyzed by ELISA, median IL-4 concentration 17.2 pg/ml, storage at −20°C before analysis) (23). Since human serum IL-4 and IL-17A are not stable during freeze-thawing, it can be posited that the absence of these in equine ACS may be attributed to their inherent instability following freezing at −80°C, a process which was applied to all ACS treatment groups (29, 36, 37). Information regarding the stability of human IFN-α in serum samples are lacking. Among the analyzed biomolecules, IFN-γ, IL-10, and CCL2 remain stable in human serum samples during freeze-thawing (29, 38, 39); for IL-1β, TNF-α, IL1-ra, CCL5, and CCL11 evidence regarding the effect of freeze-thaw cycles on their concentrations in human serum is inconclusive (29).

To the best of the author's knowledge, there has been only one previous study investigating different storage conditions for equine ACS (5). In this study, ACS of 10 horses stored at −80°C was placed at room temperature until thawing for analysis and was then re-frozen at −80°C five times. Between the five thaws, the minimum duration of freezing was 48 h. Consistent with the present study, the concentrations of IL-10, IL-1β, and TNF-α did not show any significant changes after multiple freeze-thaw cycles. However, the median concentrations for IL-10 and IL-1β were markedly higher (after two freeze-thaw cycles 60.89 and 1,657 pg/ml, respectively) and for TNF-α very markedly lower (after two freeze-thaw cycles 6.720 pg/ml) than in the present study. Regarding the IL-1β concentrations, it should be noted that IL-1β is used to induce equine synovitis *in vivo* (34, 40–43). Depending on the joint, the amount of IL-1β applied varies between 75 ng (40), 100 ng/ml (34, 41, 42), and 200 ng/ml (43). Although the median IL-1β concentrations of 1,657 pg/ml (=1.657 ng/ml) is below the concentrations used for synovitis induction, the clinical relevance may not be completely diminished, as Ross et al. (41) reduced the applied 100 ng/ml IL-1β to 50 ng/ml, due to severe lameness and effusion of the affected tarsocrural joint. The mentioned differences in the concentrations of IL-10, IL-1β, and TNF-α might be due to the already mentioned individual equine-related factors or due to differences in analysis method. ACS from healthy Thoroughbreds was analyzed in contrast to the present study that used mixed breeds and patients with orthopedic diseases. Like the present study, a bead-based multiplex assay (IL-1ra, IL-10, IL-1β, and TNF-α) was used by Hale et al. (5). Compared to conventional ELISA assays, multiplex assays have an increased sensitivity, wider dynamic quantification ranges and allow the simultaneous measurement of multiple target molecules in a single sample (33). However, unlike the present study, the fluorescent microsphere immunoassay used before included polyclonal antibodies. The detection range and specificity as well as validation data are not available, making comparisons difficult. Additionally, the study by Hale et al. (5) showed that IL-1ra levels in ACS decreased with increasing numbers of freeze-thaw cycles, being significant at the 5th thawing.

A validated equine-specific assay for IL-1ra quantification in blood products is not available. Therefore, ACS samples were analyzed by IL-1ra ELISA based on polyclonal antibodies as applied in other studies on ACS (4, 6, 7, 9–11, 19). This IL-1ra ELISA was developed to measure natural and recombinant IL-1ra in cell culture supernatant samples, while diluents in complex matrices (i.e., serum, plasma) should be evaluated prior to use in the IL-1ra ELISA, according to the manufacturer (44). Optimization experiments for sample dilutions and the buffer used as a dilution matrix were performed. Nonetheless, as ACS samples from three horses exceeded the upper detection limit of IL-1ra in this assay at the optimized dilution of 1:10, all samples were assayed again at a higher dilution (dilution 1:20). However, the resulting values were still not in the detection range for samples from two of these horses, and for the samples from the other seven horses the calculated results of the samples assayed at dilutions 10 or 20 did not agree but indicated poor inter-assay agreement, which had already been observed in preliminary experiments with five other ACS samples. Such variability and disproportionate dilution effects indicate that matrix effects interfere with the ELISA and the absolute concentrations may not be valid. Furthermore, the commercial ELISA uses bacterial expressed recombinant IL-1ra as a standard, for which the antibodies in the assay often display a different avidity than for the native equine molecule resulting in differences in titration curve slopes and errant calculation of target molecule concentrations.

Yet, the comparisons of the storage conditions can be considered, and the relative proportions of the samples in the detection range of the assay appear valid. The comparison between the samples from seven horses under the same conditions and assay dilutions did not indicate significant storage effects in agreement with the other cytokine quantifications by the more robust multiplex assays. The reliability of the exact concentrations calculated from the optical density measurement and the sample dilution warrants caution, particularly if compared between different studies. The precise identification of IL-1ra in equine ACS has been a topic of contention for the past two decades. The initial methodology employed was a human ELISA, however, the resulting data did not align with the findings obtained from mouse ELISA, which was also utilized (13). Future studies are warranted to develop a validated monoclonal antibody-based assay to detect IL-1ra levels in equine serum.

The current ACS treatment groups (TP1-80, TP1RT, TP1RF, TP2-80, TP2RT, and TP2RF) exhibited rather low levels of pro-inflammatory IFN-γ and anti-inflammatory IL-10. Notably, IFN-γ, which has not been previously measured in equine ACS, was undetectable in all ACS treatment groups from four out of 10 horses. The anti-inflammatory IL-10 has been identified in most studies investigating the composition of equine ACS. The analytical methods employed included ELISA (6, 10, 11, 19, 21) and multiplex assays (5, 9, 20). In comparison to the values recorded in this study, previous studies reported higher IL-10 concentrations. These studies included healthy horses of different breeds [unspecified adult horses (6, 9, 11), Thoroughbred horses (5), Standardbred horses (20)] ranging in age from 2 to 12 years, younger than the horses included in the present study. However, one study involving 15 stallions presented for castration exhibited markedly lower concentrations, even within the single digit nanogram range (analyzed by ELISA, assay range 0.3–20 ng/ml) (19). In the population described here, the presence of IL-10 above the detection limit was only identified in each ACS sample from two horses. This finding is consistent with the results reported by Marques-Smith et al., who observed IL-10 levels below or close to the detection limit in ACS of 19 Thoroughbred horses with spontaneous low-grade osteoarthritis (10). The IL-10 levels detected by Nakken et al. (21) in ACS of 15 healthy adult horses are also in the single-digit picogram range, however, no ELISA detection limits are provided. Despite its favorable anti-inflammatory role, the diversity in study design and detection methods do not allow draw conclusions about expectable IL-10 concentrations in equine ACS.

Declining IL-1β concentrations in ACS after storage were not robust toward Dunn's multiple comparisons test, consistent with another report of unaffected IL-1β concentrations (5). It is likely that the published IL-1β concentrations in equine ACS reflect significant interindividual differences, with a relatively wide range (median concentrations from 0 to 2,743 pg/ml) observed (4–6, 9–11, 19–21). Since IL-1β is retained as pro-protein within the cytoplasm and gains biological activity only after secretion and cleavage by caspase 1, it is unclear if employed detection methods are specific for biologically active, mature IL-1β (22). The bead-based multiplex assay used in the present study used monoclonal antibodies validated for the detection of mature equine IL-1β, thereby improving the quantification of mature IL-1β when compared to two commercial equine IL-1β ELISA kits (45).

In addition to IL-10 and IL-1β, pro-inflammatory TNF-α is commonly measured in equine ACS, too. Interestingly, previous studies reported TNF-α levels in a markedly lower range (4–6, 9–11, 19, 20). As mentioned above, this could be due to the different detection methods, including ELISA and bead-based multiplex assays. Furthermore, elevated serum levels of TNF-α have been identified in horses diagnosed with equine metabolic syndrome (EMS) or exhibiting age-related, low-grade chronic inflammation, a phenomenon known as inflamm-aging (46, 47). It should be noted, however, that none of the horses included in this study exhibited any signs of EMS or were older than 17 years of age. In contrast to its established role as a biomarker for inflammatory conditions in human medicine, TNF-α concentrations were not different in bronchoalveolar lavage fluid from healthy and asthmatic horses (48). Thus, the role of TNF-α in this context in horses remains to be elucidated. Species-specific data on the role of TNF-α as a serum marker of pro-inflammatory conditions are limited. Studies in piglets suffering from bacterial digestive diseases have shown, that serum TNF-α concentrations, in combination with other serum biomarkers, may be useful in determining the degree of intestinal histo-morphological damage (49). In the canine species, elevated TNF-α serum concentrations are associated with canine babesiosis (50) and with age, the latter providing evidence for the potential of the companion dog as a model for human aging (51).

The chemokines CCL2, CCL3, CCL5, and CCL11 belong to the CC subfamily and have yet to be elucidated in equine ACS samples. In the present study, they were detected in all ACS treatment groups from each horse. Chemokines, also known as chemotactic cytokines, form a complex network that includes many ligands and receptors, as well as regulatory proteins involved in overlapping and diverse cellular processes (52). CCL2, CCL3, CCL5, and CCL11 have been classified as pro-inflammatory acting chemokines based on their functions in humans (53). CCL2, CCL3, CCL5, and CCL11 were shown to be secreted by equine blood leukocytes stimulated *in vitro* (54). However, chemokines in general have only been minimally studied in equine joint-associated diseases. In an equine IL-1β-induced carpal synovitis model, CCL2, CCL3, CCL5, and CCL11 were identified as sensitive biomarkers during the early stages of joint inflammation (synovitis), exhibiting temporal variations in their response (34). In contrast, differences between the synovial fluid levels of CCL2 and CCL5 in equine osteoarthritis (OA)-affected or healthy joints were not reported (55). Furthermore, most synovial fluid samples from equine OA-affected and healthy joints did not reveal detectable CCL2, and CCL11 was shown to be significantly reduced in synovial fluid from OA-affected joints when compared to synovial fluid from healthy joints (55). As it is assumed that the entirety of active biomolecules is responsible for the positive clinical effects of OA therapy, the relevance of the chemokines studied cannot be conclusively assessed. Presumably, since the cell-free ACS reflects the predominantly monocyte-derived blood cell secretome, chemokines may allow to orchestrate potential migrating immune cells that are beneficial for therapeutic purposes. However, among the chemokines selected for analysis, CCL5 is secreted by T lymphocytes *in vitro* without stimulation in short time. This may indicate that blood cells other than monocytes contribute their secretome to ACS.

Cytokine and chemokine levels in ACS might be correlated to abnormal values identified in the blood chemistry as in the case of equine platelet concentrate and platelet lysate (56). However, a recent study did not find an association between hematology or biochemistry analyses and ACS levels of IL-10, IL-1ra, IL-1β, TNF-α, IGF-1, and TGF-β (19). The current study did not evaluate horses' hematology or biochemistry.

The present study is limited by the small sample size, which included only female horses that met the inclusion criteria. Patient variables related to venipuncture for ACS preparation, such as time, relationship to potential surgery, sedation or other potentially influencing factors, were not standardized. In addition, ACS treatment groups reflect selected storage conditions which may not be the only clinically relevant conditions. The biomolecules analyzed were also limited by the restricted availability of validated equine-specific detection methods. The current study is further limited using only one medicinal product for the preparation of ACS.

Considering the interindividual differences observed, it is warranted to conduct comprehensive screening studies to ascertain reliable biomolecule concentrations of equine ACS. In addition, the lack of significant differences in biomolecule concentrations between the selected storage conditions may be due to the large interindividual differences and the relatively high number of statistical tests performed, thus reducing the power of the conclusions. Furthermore, the current study did not include a non-stored control sample, which would have enabled the detection of biomolecules that were unstable during freezing at −80°C. This was not possible as sample collection and analysis were performed in different countries. Nevertheless, analysis was done at an external laboratory facility and the immediate application of ACS without any storage period may be less pertinent in a clinical context.

Cytokines and chemokines as detected in ACS represent components of the endogenous molecular network that regulate immunological interactions. It is likely that the totality of cytokines and chemokines but also other bioactive molecules such as extracellular vesicles or microRNA, is of therapeutic value as a patient-specific immunological signature.

The present study can conclude that the practical storage of ACS (Orthokine^®^ vet irap 10 ml; ORTHOGEN Veterinary GmbH; ACS preparation according to manufacturer's instructions) for 6 h at room temperature or in a refrigerator immediately (TP-80, TP1RT, TP1RF) after production or after 7 months (28 weeks) of storage at −20°C (TP2-80, TP2RT, TP2RF) does not lead to significant changes in the concentration of selected cytokines and chemokines. However, the concentration of most of the biomolecules analyzed showed clear interindividual differences. This may partly explain the lack of evidence for consistent beneficial effects of equine ACS *in vitro* and *in vivo*. ACS is an equine patient-specific biological therapeutic agent without a standardized composition and therefore it may also contain signaling molecules that prevent a clear therapeutic effect in individual cases. The present study does not allow to draw conclusions regarding the clinical effects of the cytokines and chemokines under investigation.

## Data Availability

The original contributions presented in the study are included in the article/Supplementary material, further inquiries can be directed to the corresponding author.
