# Supplementary material for: Selected cytokine and chemokine concentrations in equine autologous conditioned serum are similar under defined and practically relevant storage conditions
Source: Front Vet Sci. 2025 May 27;12:1588240. doi: 10.3389/fvets.2025.1588240 (PMC12150348; doi:10.3389/fvets.2025.1588240)
Supplement: Supplementary file 1 [file Table_1.docx]

Supplementary Tables

**Table S1.** Overview of patient data (*n*=10) including age in years, breed, bodyweight in kilogram, sex, indication and application site for autologous conditioned serum (ACS). In 10 adult horses that were presented for a therapeutic ACS application, additional 6 ACS samples were prepared and labelled with respect to their storage treatment as the following treatment groups: TP1-80 [time point 1: immediate storage at -80 °C]; TP1RT [time point 1: 6 hours storage at room temperature]; TP1RF [time point 1: 6 hours storage at refrigerator temperature]; TP2-80 [time point 2: immediate storage at -80 °C]; TP2RT [time point 2: 6 hours storage at room temperature]; TP2RF [time point 2: 6 hours storage at refrigerator temperature]). Time point 1 was set immediately after ACS preparation, and time point 2 was set after a 7 month (28 weeks) of storage at -20 °C and thawing in a water bath (37 °C). After the storage testing was completed on treatment groups from the 10 horses, all ACS samples underwent an initial slow thaw process for re-aliquoting and were again stored at -80 °C. All 6 ACS samples per horse were analyzed by multiplex assays validated for the equine mediators TNF-α, IL-1β, IFN-γ, IL-17A, IL-4, IL-10, IFN-α, CL2, CCL3, CCL5, CCL11.

| **No.** | **Age** [years] | **Breed** | **Bodyweight** [kg] | **Sex** | **Indication and application site for autologous conditioned serum (ACS)** |
| --- | --- | --- | --- | --- | --- |
| **1** | 9 | Hanoverian | 480 | female | **osteoarthritis (OA) right metacarpophalangeal joint**   - chronic synovitis, partial-thickness cartilage erosion of the metacarpus, partial pannus formation of the metacarpus, several free and one adherent intraarticular fragment - intraarticular application 2 days after arthroscopy |
| **2** | 3 | Oldenburger | 550 | female | **acute vertical perforating laceration at the dorsolateral aspect of the right metacarpophalangeal joint**   - acute synovitis locally at joint capsule laceration; synovial fluid cloudy, white blood cell count (WBCC) 13.8 G/l, polymorphonuclear cells (PMN) 62 %, active macrophages 35 %, total protein (TP) 46.9 g/l, glucose 1.39 mmol/l; bacterial isolation negative - intraarticular application 5 days after arthroscopy |
| **3** | 17 | Hanoverian | 583 | female | **subacute desmitis of the inferior check ligament of the left front limb**   - thickening of the inferior check ligament on its entire length, inhomogeneous structure and irregular contour, several circumscribed hypoechogenic areas - intralesional application |
| **4** | 3 | Hanoverian | 421 | female | **acute V-shaped perforating laceration at the plantar aspect of the right digital flexor tendon sheath at the level of the metatarsophalangeal joint**   - acute synovitis - intrathecal application 6 days after tenovaginoscopy |
| **5** | 14 | Icelandic Horse | 350 | female | **subacute perforating laceration (3 cm x 4 cm) at the plantar aspect of the right digital flexor tendon sheath at the level of the proximal interphalangeal joint**   - digital flexor tendon sheath markedly distended and increasingly warm, acute synovitis with deposition of fibrin clots and adhesion formation; synovial fluid cloudy, white blood cell count (WBCC) 65.5 G/l, polymorphonuclear cells (PMN) 88 %, active macrophages 9 %, total protein (TP) 58.8 g/l, glucose 3.75 mmol/l; bacterial isolation negative - intrathecal application 3 days after tenovaginoscopy |
| **6** | 11 | Hanoverian | 580 | female | **acute proximal suspensory desmitis left hindlimb**   - nerve block of the left deep branch of the lateral plantar nerve positive, proximal part of the suspensory ligament at the medial aspect thickened without distance to the cortical surface of the third metatarsal bone, structure of the proximal suspensory ligament with a single circumscribed hypoechogenic lesion during on- and off-beam - intralesional application |
| **7** | 14 | Saxon-Thuringian Heavy Warmblood | 694 | female | **subacute desmitis of the inferior check ligament of the right front limb**   - structure of the inferior check ligament on its entire length inhomogeneous and loosened, during longitudinal views loss of directional fiber course, several circumscribed hypoechogenic areas - intralesional application |
| **8** | 15 | German Sport Horse | 570 | female | **chronic osteoarthritis (OA) of the right talocrural joint**   - marked synovitis, marked cartilage erosions of the talus with partial chondromalacia - intraarticular application 4 days after arthroscopy |
| **9** | 5 | Haflinger | 477 | female | **acute lesion of the lateral margin of the proximal manica flexoria at the left hind limb**   - intrathecal block of the digital flexor tendon sheath positive, during weight-bearing and non-weight-bearing contour interruption of the lateral aspect of the proximal manica flexoria - intrathecal application 3 days after tenovaginoscopy |
| **10** | 16 | Icelandic Horse | 407 | female | **acute primary plantar annular ligament syndrome at the right hind limb**   - marked as well as dolent and increasingly warm distension of the digital flexor tendon sheath, distal flexion test markedly positive, low 6-point block positive, subcutaneous tissue at the plantar aspect of the fetlock region thickened and difficult to distinguish from the adjacent plantar annular ligament and the superficial digital flexor tendon (SDFT), contour of the SDFT with mild fibrillations, palmar annular ligament medial 2.1 mm and lateral 1.8mm, palmar annular ligament plantarocentrally not measurable - intrathecal application and local plantar infiltration at the level of the plantar annular ligament |

**Table S2. Median (interquartile range) concentrations of cytokines and chemokines in autologous conditioned serum (ACS).** TP1-80 (time point 1: immediate storage at -80 °C) is the basal ACS control, TP1RT (time point 1: 6 hours storage at room temperature) was stored at room temperature for 6 hours, TP1RF (time point 1: 6 hours at refrigerator temperature) was stored at refrigerator temperature at 6-8 °C for 6 hours; treatment groups TP2-80, TP2RT, and TP2RF were stored at -20 °C for 7 month (28 weeks) (time point 2) before they were thawed at a water bath (37 °C), and then either immediately were stored at -80 °C (TP2-80) or were stored for 6 hours either at room temperature (TP2RT) or at refrigerator temperature at 6-8 °C (TP2RF). CCL2 concentration values of 1,000,000.00 pg/ml indicate a measured value that was above the upper limit of detection (> 140 000 pg/ml).

|  | **TP1-80** | **TP1RT** | **TP1RF** | **TP2-80** | **TP2RT** | **TP2RF** |
| --- | --- | --- | --- | --- | --- | --- |
| **IL-1β**  [pg/ml] | 32.50  (0 – 800.75) | 0  (0 – 477.25) | 84.00  (0 – 889.50) | 0  (0 – 174.75) | 0  (0 – 8.25) | 0  (0 – 245.25) |
| **TNF-α** [pg/ml] | 19,661.50  (7,401.25 – 23,859.25) | 16,206.00  (7,004.50 – 21,345.00) | 21,413.50  (8,753.25 – 27,100.00) | 15,268.50  (8,115.75 – 20,793.50) | 22,687.50  (10,870.75 – 24,391.25) | 17,229.00  (8,586.50 – 20,300.50) |
| **IFN-γ**  [U/l] | 10.50  (0 – 19.25) | 0  (0 – 19.25) | 11.50  (0 – 19.25) | 12.00  (0 – 22.25) | 10.00  (0 – 16.75) | 11.00  (0 – 18.75) |
| **IL-10**  [pg/ml] | 12.00  (0 – 17.50) | 5.00  (0 – 17.25) | 6.5  (0 – 17.25) | 10.50  (0 – 15.00) | 7.00  (0 – 17.00) | 6.50  (0 – 14.25) |
| **CCL2**  [pg/ml] | 98,812.50  (44,413.25 – 1,000,000.00) | 546,061.00  (43,708.25 – 1,000,000.00) | 84,645.00  (44,545.50 – 1,000,000.00) | 544,406.50  (42,638.00 – 1,000,000.00) | 88,043.50  (44,605.25 – 1,000,000.00) | 92,088.00  (45,161.75 – 1,000,000.00) |
| **CCL3**  [pg/ml] | 42,049.00  (26,203.50 – 60,484.75) | 41,243.00  (27,604.25 – 60,783.50) | 45,8806.00  (23,588.50 – 58,166.00) | 42,471.00  (25,187.25 – 61,322.75) | 36,020.00  (24,461.00 – 56,535.50) | 42,858.00  (26,313.75 – 60,428.00) |
| **CCL5**  [pg/ml] | 263.50  (137.50 – 439.75) | 281.00  (114.25 – 414.25) | 273.00  (130.00 – 436.75) | 256.50  (129.50 – 376.00) | 266.50  (126.50 – 375.25) | 264.00  (123.25 – 401.75) |
| **CCL11**  [pg/ml] | 9,001.00  (4,329.50 – 14,180.50) | 8,983.00  (4,361.25 – 13,958.75) | 8,818.50  (4,203.75 – 15,331.50) | 8,493.50  (4,178.00 – 11,738.50) | 8,738.50  (4,172.25 – 11,465.25) | 8,201.50  (4,366.75 – 11,796.75) |
